# Magnetic Resonance Spectroscopy in Depressed Subjects Treated With Electroconvulsive Therapy—A Systematic Review of Literature

**DOI:** 10.3389/fpsyt.2021.608857

**Published:** 2021-03-25

**Authors:** Vera Jane Erchinger, Lars Ersland, Stein Magnus Aukland, Christopher C. Abbott, Leif Oltedal

**Affiliations:** ^1^Department of Clinical Medicine, University of Bergen, Bergen, Norway; ^2^Department of Clinical Engineering, Haukeland University Hospital, Bergen, Norway; ^3^Department of Biological and Medical Psychology, University of Bergen, Bergen, Norway; ^4^NORMENT Centre of Excellence, Haukeland University Hospital, Bergen, Norway; ^5^Department of Psychiatry, University of New Mexico School of Medicine, Albuquerque, NM, United States; ^6^Department of Radiology, Mohn Medical Imaging and Visualization Centre, Haukeland University Hospital, Bergen, Norway

**Keywords:** depression, electroconvulsive therapy, magnetic resonance spectroscopy [(1)H MRS], neurotransmitters, brain

## Abstract

Electroconvulsive therapy (ECT) is considered to be the most effective acute treatment for otherwise treatment resistant major depressive episodes, and has been used for over 80 years. Still, the underlying mechanism of action is largely unknow. Several studies suggest that ECT affects the cerebral neurotransmitters, such as gamma-aminobutyric acid (GABA) and glutamate. Magnetic resonance spectroscopy (MRS) allows investigators to study neurotransmitters *in vivo*, and has been used to study neurochemical changes in the brain of patients treated with ECT. Several investigations have been performed on ECT-patients; however, no systematic review has yet summarized these findings. A systematic literature search based on the Prisma guidelines was performed. PubMed (Medline) was used in order to find investigations studying patients that had been treated with ECT and had undergone an MRS examination. A search in the databases Embase, PsycInfo, and Web of Science was also performed, leading to no additional records. A total of 30 records were identified and screened which resulted in 16 original investigations for review. The total number of patients that was included in these studies, ignoring potential overlap of samples in some investigations, was 325. The metabolites reported were N-acetyl aspartate, Choline, Myoinositol, Glutamate and Glutamine, GABA and Creatine. The strongest evidence for neurochemical change related to ECT, was found for N-acetyl aspartate (reduction), which is a marker of neuronal integrity. Increased choline and glutamate following treatment was also commonly reported.

## Introduction

Depression is a common and debilitating psychiatric disorder. According to the World Health Organization, more than 264 million people suffer from depression globally (1). A depressive episode can be mild, moderate or severe, depending on the number of symptoms and their severity, and can be part of a unipolar disorder, such as a recurrent depressive disorder or bipolar affective disorder when accompanied by hypomania or mania (1). Electroconvulsive therapy (ECT) has been available for more than 80 years and is still considered the most effective treatment for major depressive episodes. Up to 70–90% of patients respond (>50% symptom reduction) to the treatment in controlled trials (2, 3) and corresponding high response rates have been found in clinical practice (4). It is in most cases more effective than pharmacological treatment (5), and an alternative to the 30% of patients that do not respond to pharmacotherapy, even after several treatment steps (6).

However, the mechanism of action behind this treatment is largely unknown. The lack of understanding may have caused more skepticism to ECT than the treatment effect would suggest. ECT treatment consists of electric stimulation delivered through scalp electrodes while patients receive general anesthesia. The electrical field from the stimulation excites neurons past their threshold to release neurotransmitter and hereby triggers an epileptic seizure, normally lasting 14–45 s. Muscular contraction is avoided by use of drugs that cause neuromuscular blockade. Typically, ECT is administrated 2–3 times a week for 3–6 weeks, but administration may vary from site to site and with indication. The two most common ways of electrode placement are the bitemporal (BT) placement and the right unilateral placement (RUL) (7). Cognitive side effects impacting memory and other cognitive domains are unfortunately common and add to the stigma of the procedure (8). The ECT-mediated cognitive impairment can last for weeks after an ECT series and slow the recovery from functional impairment related to a depressive episode (9, 10). Investigations focused on ECT's mechanism of action should also account for and disentangle brain changes associated with the cognitive sequalae.

**Figure 1:**
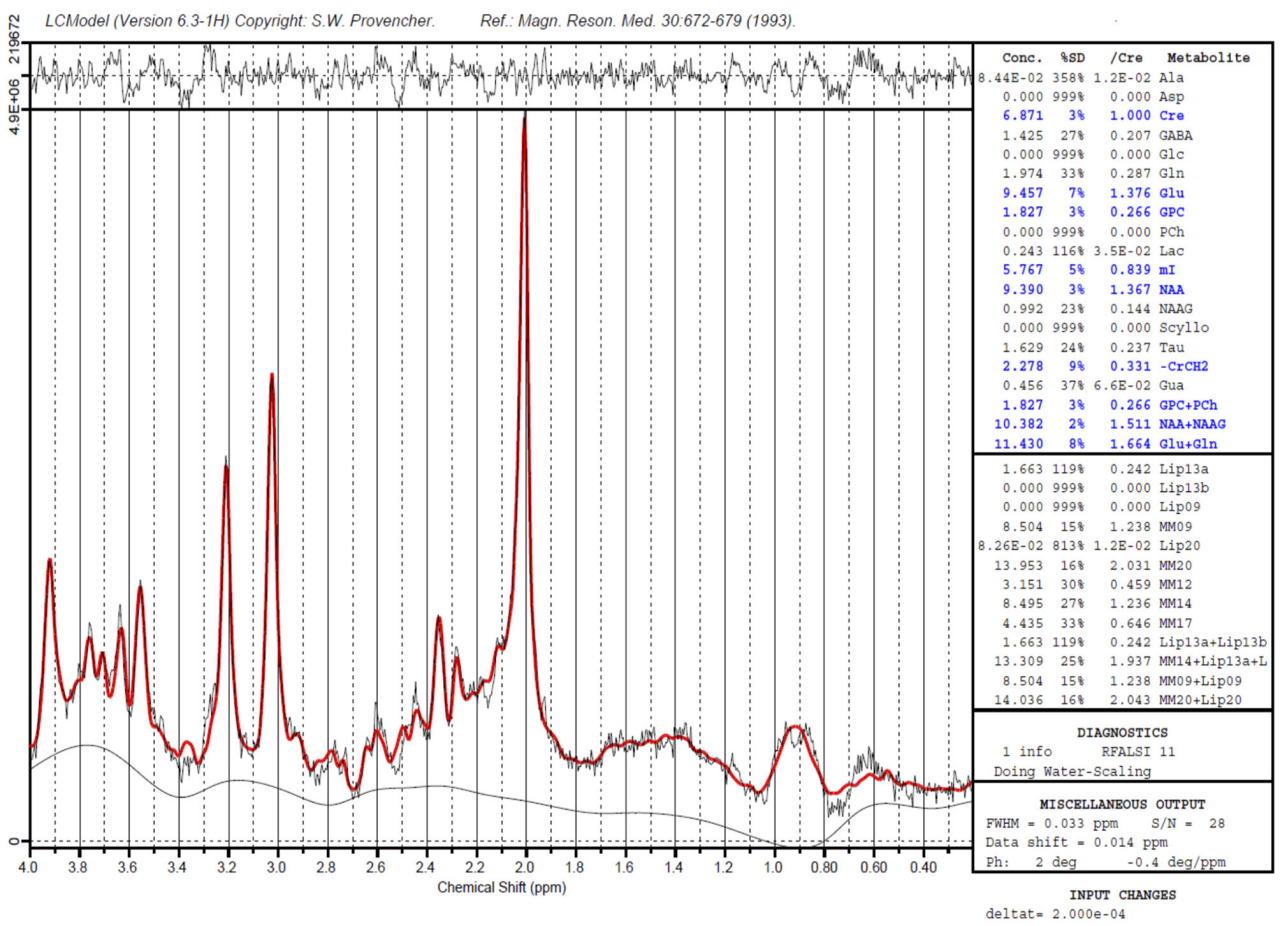
A sample H^1^- MRS spectrum, created with the LCModel software. Peaks of Creatine (two peaks), myo-Inositol, Choline and N-acyl aspartate are marked. The chemical shift (ppm) is shown on the X-axis. The Y- axis shows the intensity of the signal (measured in institutional units). The area under the curve is proportional with the concentration of the metabolite. For GABA, a special technique is required.

For the last 3 decades hydrogen magnetic resonance spectroscopy (H-MRS) has helped explore the biochemical aspects of brain changes (11). MRS takes advantage of protons reacting slightly different to the magnetic field, depending on their chemical environment. This makes it possible to distinguish between different chemical compounds, and to quantify their concentrations, even at fairly low concentrations. The metabolite is measured in a specified volume, the voxel. The nuclear spin of certain nuclei is measured in the voxel, and will upon manipulation give a resonance at a certain frequency, characteristic for a certain metabolite. The most common nucleus used in MRS is the hydrogen proton (H^1^), but phosphorus (^31^P) is another nucleus that can be measured. Figure 1 is illustrative for a typical H^1^-MRS spectrum, made with the LCModel software (12).

MRS gives the opportunity to study a broad array of compounds *in vivo* in a non-invasive way. The two main methods for H^1^-MRS are point resolved spectroscopy, PRESS and stimulated echo acquisition mode, STEAM. Challenges in MRS are overshadowing of metabolites that are present in very small amounts by other largely abundant substances. For gamma-aminobutyric acid (GABA) this is the case, and a special technique is required (13, 14). MRS of the brain is primarily used for research purposes, but is also used clinically, in newborns with asphyxia, both in evaluation of the hypoxic-ischemic injury and as a predictor of outcome (15, 16). MRS is also included in the work-up of brain tumors, in some centers as a routine exam, while in others as a supplementary tool, as it may help in grading the tumor, decide the extension of the tumor and as a guidance to biopsy (17). Current research will hopefully encourage further adaptation of the method for clinical use in fields such as psychiatry and ECT- even if current reviews so far have concluded with conflicting results (18).

## Materials and Methods

**Figure 2:**
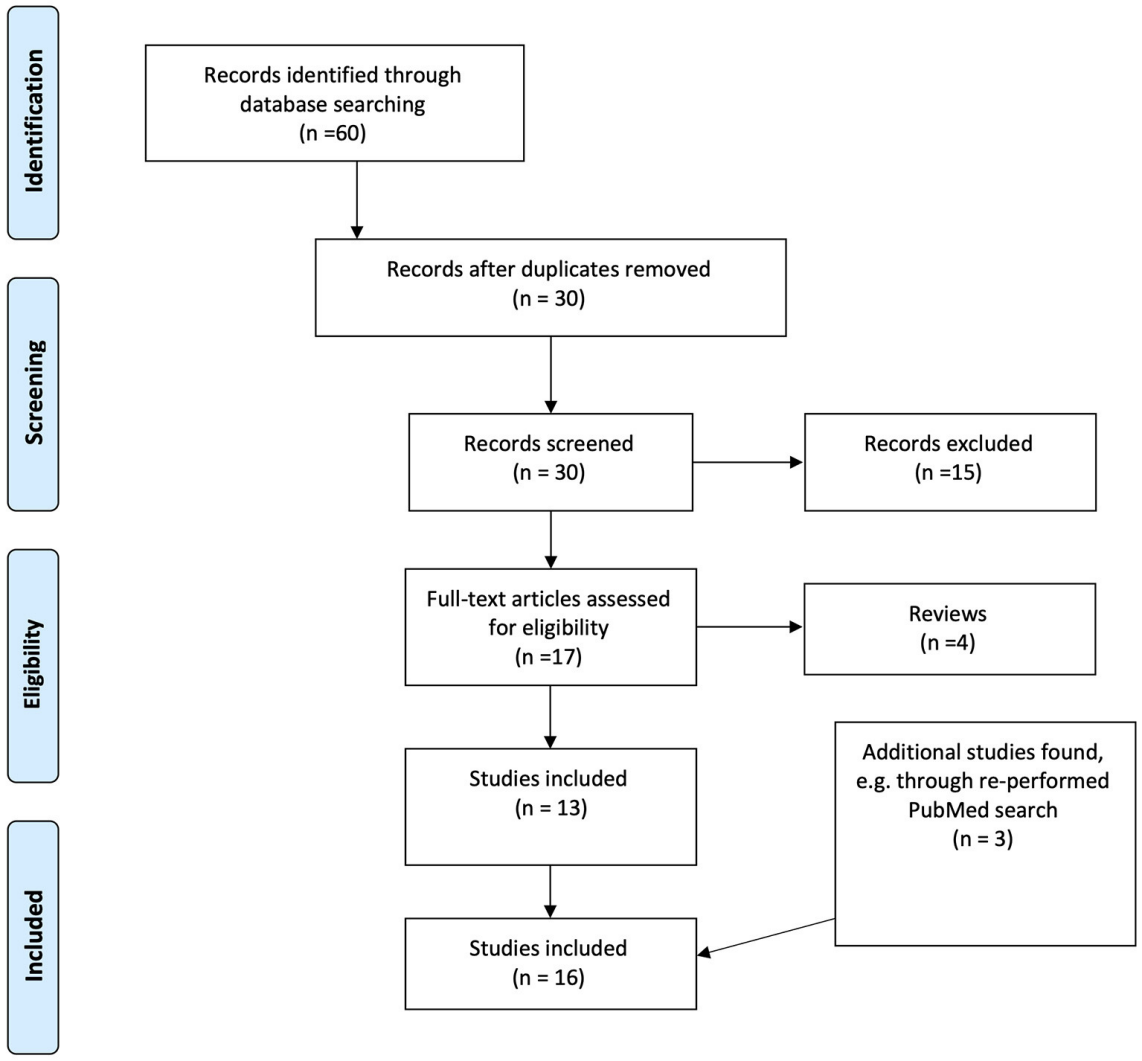
Flow chart of the results of the literature search and inclusion process, based on the PRISMA flowchart.

In preparation for this review a structured literature search was performed in the databases PubMed (25 records), Embase (51 records), PsycInfo (16 records), and Web of Science (73 records) in December 2016. After exclusion of non-relevant investigations and duplicates 25 investigations remained, all of which could be found by using PubMed. Hence further searches were performed in PubMed with the following criteria: “(Depression OR depressive disorder) AND (electroconvulsive therapy) AND (magnetic resonance spectroscopy)” (31 records) and “(Major depression) AND (electroconvulsive therapy) AND (Magnetic resonance spectroscopy)” (29 records). All investigations on animals were excluded, as well as records that were abstracts, publications on children and adolescents, investigations that did not use H^1^-MRS and investigations reporting manual comparisons of spectra. After exclusion 17 investigations remained, of which 13 were original investigations included for this review. References of all investigations were checked for additional relevant investigations. The search was last performed on the 15th of December 2020. Three additional investigation had been published. The literature search through PubMed is described based on the PRISMA flowchart (19), see Figure 2.

## Results

**Figure 3:**
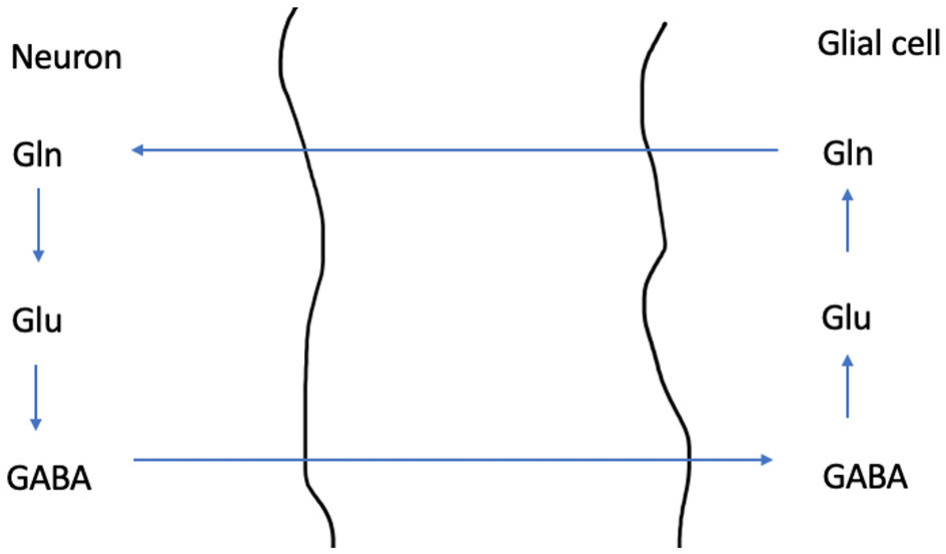
A schematic overview illustrating the Glutamine - Glutamate- GABA cycle. GABA is the most common inhibitory neurotransmitter in our nervous system, while Glutamate is the most common excitatory neuro transmitter. Cells where glutamate is synthetized from glutamine are glutamatergic, while cells where this pathway ends in synthesis of GABA are GABAergic. Glutamine is synthetized in the glial cell, and is then transported into the neuron. In the neuron's mitochondria glutamate is synthetized from glutamine. Then glutamate is stored in synaptic vesicles (not shown). Alternatively, GABA can be synthetized from glutamate.

Our systematic search resulted in 16 original investigations which were included for review, see Table 1. An overview of the various metabolites which were investigated across all included investigations, together with their described physiological functions is given in Table 2. A schematic simplified overview of the glutamine-glutamate- GABA cycle is given in Figure 3, for illustration of how these neurotransmitters relate [adapted from (35, 36)].

**Table 1:** Overview of all original investigations that were included in the systematic review.

**References**	**Number of** **patients (M)/** **controls (M)**	**Age of** **patients (SD)/** **controls (SD)**	**Diagnosis**	**MR scan** **times patients**	**MR scan** **times controls**	**ECT electrode** **placement**	**Scanner** **strength**	**ROI/voxel** **placement**	**Type of** **spectroscopy**	**Metabolites** **reported**	**Difference at** **baseline, compared to** **controls**	**Metabolites with** **significant increase** **in patients**	**Metabolites with** **significant decrease** **in patients**
Knudsen et al. (20)	11 (5)/11 (5)	38.4 (10.9)/38.8 (10.8)	MDD or BP depressive episode	(1) 1–2 days before ECT, (2) after ECT series	(1) One time point only	RUL	3 T	(1) PFC, (2) OCC	MEGA-PRESS	GABA, NAA, Cho, Cre, Glu, Gln, GSH			PFC: NAA/Cr (*p* = 0.08)^a^
Cano et al. (21)	12 (6)/10 (5)	59.2 (8.0)/54.4 (8.4)	TRD	(1) 24–48 h before ECT, (2) 24–48h after first ECT, (3) 24–48 h after 9th ECT, (4) 2 weeks after completion of ECT	Twice, 5 weeks apart	BL	3 T	Left hippocampus	PRESS	NAA, Glx, Cho, Cre		Glx/Cre (trend)	NAA/Cre (*p* = 0.015)
Njau et al. (22)	50 (23)/33 (14)	43.8 (14)/39.3 (12)	Major depressive episode in unipolar or bipolar depression	(1) 24 h before ECT, (2) between 2nd and 3rd ECT, (3) within 1 week of finishing ECT	Twice, 4 weeks apart	RUL	3 T	(1) dACC, (2) sgACC, right and left hippocampus	PRESS	NAA, Cho, Glx, Cre	Left hippocampus: reduced NAA (*p* = 0.001) and elevated Glx (*p* = 0.049), sgACC: decreased Glx (*p* = 0.025)	dACC: Cre (*p* = 0.002), sgACC: Glx (*p* = 0.05), Cre (*p* = 0.010)	dACC: NAA (*p* = 0.003), left hippocampus: Glx (*p* = 0.003), right hippocampus: NAA (*p* = 0.002)
Njau et al. (23)	50 (19)/33 (14)	43.8 (14)/39.3 (12)	MDD	(1) 24 h before ECT, (2) between 2nd and 3rd ECT, (3) within 1 week of finishing ECT	Twice, 2–5 weeks apart	BL/RUL	3 T	(1) ACC, (2) hippocampus	PRESS	mI		ACC: mI [T1–3 (*p* = 0.02) and T2–3 (*p* = 0.03)]	
Jorgensen et al. (24)	19 (5)/0	52.3 (11.5)	Unipolar or bipolar severe depression	(1) Before ECT, (2) 1 week, and (3) 4 weeks after ECT-series		16 BL, 3 RUL	3 T	Hippocampus, left and right	PRESS	Glu, mI, NAA, GPC + PCh, Cr + PCr, Glx		mI (*p* = 0.033)^b^, Cr + PCr (*p* = 0.015)^b^	NAA (*p* = 0.031)^b^
Zhang et al. (25)	10 (4)/10 (4)	44 (7.9)/39.0 (9.6)	MDD	(1) Baseline, (2) after 2nd ECT, (3) after 6th ECT	Twice, 2 weeks apart	?	3 T	Midline ACC	PRESS	Glu, NAA, Cho	Lower Glu in patients (*p* < 0.01)	Glu (*p* > 0.037), Cho (*p* > 0.047)	NAA (*p* < 0.48)
Merkl et al. (26)	25 (2)/25 (3)	51.76 (13.16) for responders, 46.38 (12.93) for nonresponders /36.30 (13.98)	MDD	(1) Baseline, (2) after 9 ECTs, (3) at completion of ECT	Same as patients?	2 BL, 25 RUL	3 T	(1) DLPFC, (2) ACC	PRESS	Cho, Cre, NAA, Glu	ACC: NAA (*p* = 0.005) and Glu lower (*p* > 0.001)	ACC: NAA (*p* = 0.003), responders only	DLPFC: NAA (*p* = 0.04)
Michael et al. (27)	28 (10)/28 (10) included both MDD and BP	59.7 (15.2) for MDD, 54.1 (16) for BP/58.5 (8.9) for MDD, 52.2 (14.4) for BP	Major depressive episode. MPP or BP	(1) Before ECT, (2) after ECT	?	27 RUL, 1 mixed	1.5 T	Amygdala and hippocampus	STEAM	NAA, Cho, Cre, Glx	Reduction of Glx in unipolars only, compared to controls (*p* = 0.049)	NAA (*p* = 0.02), Glx (*p* = 0.049), both in responders only	Glx (*p* = 0.04), unipolar depression only
Michael et al. (28)	12 (4)/12 (6)	63.4 (10.6)/62 (8.7)	Severe depressive disorder	(1) 1–2 days before, ECT (2) 1–3 days after ECT	?	?	1.5 T	Left DLPFC	STEAM	NAA, Glx, Cho, Cre	Lower Glx in patients (*p* < 0.002)	glx (*p* = 0.02) (responders)	
Sanacora et al. (29)	8 (5)/0	46 (5.3)	Major depression	(1) Before ECT, (2) after ECT		7 BL, 1 UL	2.1 T	OCC	MEGA-PRESS?	GABA		GABA (*p* < 0.02)	
Pfleiderer et al. (30)	17 (5)/17 (5)	61 (11.2)/60.1 (10.9)	Recurrent unipolar major depressive disorder	(1) before ECT, (2) after ECT	One timepoint only	15 UL, 2 BL	1.5 T	ACC	STEAM	Glx, NAA, Cho, Cre	Reduced Glx compared to healthy controls (*p* < 0.0001)	Glx (*p* = 0.04), in responders	
Obergriesser et al. (31)	12 (4)/0	63.8 (14.3)	Major depressive episode	(1) Before ECT, (2) 12–32 months after ECT		?	1.5 T	Hippocampus left and right	PRESS	NAA, Cre, Cho		Cr (*p* = 0.02), Cho (p=0.001)	
Ende et al. (32)	17 (7)/2 4(2) and 6 (4) remitters as control group	61.3 (13.4)/35.3 (11.6) and 49.7 (13.4)	?	(1) Before, (2) after 5 or more ECTs	?	14 RUL, 3 BL	1.5 T	Hippocampus	PRESS	NAA, Cre, Cho		Cre (*p* = 0.02), Cho (*p* = 0.001)	
Erchinger et al. (33)	41 (19)/35 (1323)	53 (16)/53 (14)	Major (unipolar or bipolar) depressive episode	(1) before, (2) after ECT	Two, similar time interval as patients	41 RUL with switch to BL in 8	3 T	ACC	MEGA-PRESS	GABA			
Tosun et al. (34)	13 (6)/14 (7)	45.9 (10.9)/41.1 (9.3)	Major depressive disorder	(1) before, (2) after 6th ECT	One timepoint only	BL	3 T	ACC	PRESS	NAA, Cho, Cre, NAA/Cre, Cho/Cre			NAA/Cr (*p* < 0.001)

*M, male; SD, standard deviation; ECT, electroconvulsive therapy; RUL, right unilateral electrode placement; BL, bilateral electrode placement; UL, unilateral (side not specified in publication); ROI, region of interest; ACC, anterior cingulate cortex; dACC, dorsal anterior cingulate cortex; sgACC, subgenual anterior cingulate cortex; STEAM, stimulated echo acquisition mode; PRESS, point resolved spectroscopy; MDD, major depressive disorder; BD, bipolar disorder; TRD, treatment resistant depression; PFC, prefrontal cortex; DLPFC, dorsolateral prefrontal cortex; OCC, occipital cortex; GABA, gamma-aminobutyric acid; NAA, n-acetyl aspartate; Cr, creatine; Glx, glutamate and glutamine combined; Glu, glutamate; Gln, glutamine; Cho, choline; mI, myoinositol; GPC + PCh, glycerophosphorylcholine + phosphorylcholine; Cr + PCr, creatine + phosphorcreatine; GSH, glutathione*.

a *Effect size 1.18*.

b *Significance level set to p < 0.01 to correct for multiple analyses*.

**Table 2:** An overview of names, and supposed role/function of the most common substances investigated in this report.

**Substance**		**Putative function/role**	**No of studies** **included in review**
**Abbreviation**	**Full name**		
NAA	N-acetyl aspartate	Neuronal marker, takes part in biosynthesis of myelin, marker of formation and maintenance of myelin.	11
Cho	Choline	Biomarker for status of membrane phospholipid metabolism. High levels of choline = high levels of membrane turnover.	8
mI	myo-Inositol	A sugar involved in regulation of neuronal osmolarity. Traditionally a marker of glial cells, newer theory suggests that glial cells are a storage for mI.	2
Glu, Glx	Glutamate, glutamine+ glutamate	The most abundant excitatory neurotransmitter. Glu has been implicated in depression.	8
GABA	gamma aminobutyric acid	The most abundant inhibitory neurotransmitter. See Figure 3. Accumulating evidence suggests role in depression. SSRI increase GABA.	2
Cre	Creatine	Marker of energy metabolism. Is supposed to be relatively stable, and is often used as reference metabolite.	8

*The column to the right shows how many of the included investigations that measured a certain metabolite*.

### N-Acetyl Aspartate

Eleven investigations reported pre- vs. post-treatment comparisons of NAA values. Michael et al. (27) reported an *increase* of NAA in the amygdala/hippocampus region, but this was significant only for patients that responded to ECT. Merkl (26) reported a *reduction* in NAA in the dorsolateral prefrontal cortex, but an *increase* in the ACC. Knudsen (20), Cano (21) and Njau (22) and Zhang (25) all report *reduced* NAA after a course of ECT-treatment, measured in the prefrontal cortex (PFC), left hippocampus and anterior cingulate cortex (ACC) and hippocampus, respectively. Tosun (34) also reported reduced NAA in the ACC; however as creatine was used for reference, the authors discuss that his was due to a relative change in creatine. Only two studies, Njau (22) and Merkl (26), report significantly *lower* levels of NAA for patients compared to a healthy control group at baseline. Four of the published investigations reported *no significant changes* in NAA: Jørgensen (24), Pfleiderer (30) and Obergriesser (31) (long time follow up) and Ende (32). For an overview of which investigations used creatine ratio, see Table 1.

### Choline

Nine investigations reported choline. Obergriesser (31), Ende (32), and Zhang (25) all reported significant *increase* of choline, measured in the hippocampus (Obergriesser, Ende) or ACC (Zhang). In Obergriesser (31) the increased choline values returned to pre-treatment values in all but one patient in post-treatment follow up. Other investigations *did not have significant findings* (21, 26, 27, 30, 34), or did not report findings (28).

### Myoinsitol

Njau et al. (23) report significantly *increased* mI in the dorsal ACC after ECT, while measurements from the hippocampal voxel showed no significant change in mI. Jorgensen et al. (24) did not find a significant change of mI when measured in the hippocampi bilaterally.

### Glutamate/Glutamine

Several previous studies on depression report reduced glutamate or glutamate and glutamine combined (Glx) prior to treatment in depressed subjects (37). Consistent with this, prior to treatment: Merkl (26) and Zhang (25) do report *lower* values for glutamate in patients (before treatment) compared to controls. Njau (22), Pfleiderer (30) and Michael (28) report *lower* values of Glx in patients (before treatment) compared to controls. Michael (27) reports reduced Glx, but only in unipolar depressed subjects compared to controls. This finding was not significant, but trending, when including patients suffering from bipolar disorder. After treatment, Njau (22) reports a significant increase of Glx in the ACC. A similar result was reported by Zhang (25) for glutamate. Pfleiderer (30) reports increased Glx only in patients that responded to treatment. Michael et al. (28) reports increased Glx- levels in the dorsolateral prefrontal cortex (DLPFC), significant for responders only, but published on partly the same material as Pfleiderer et al. (30).

### Gamma-Aminobutyric Acid

Sanacora et al. (29) reported increase in occipital GABA after treatment in 8 patients. However, there was no significant correlation between treatment response and increase in GABA. In 2018 Knudsen (20) replicated the study, adding a healthy control group, but could not find any significant changes in GABA- levels in relation to ECT. Knudsen (20) added a voxel in the DLPFC, but could not find any significant changes in GABA levels in this area either. In a recent study which included 41 patients from two independent sites, Erchinger et al. (33) reported no changes in GABA levels in the ACC after ECT, however a link to reduced performance on the effortful cognitive processing was found in exploratory analysis.

### Creatine

Eight investigations report having measured creatine. Njau (22) reports a significant increase in creatine after treatment in the ACC and Ende (32) reported a significant increase measured in the hippocampus. Other investigations did not report significant findings (24, 26, 27, 30, 31, 34).

### 1.28 ppm Peak

Manganas et al. (38) reported a 1.28 ppm peak *in vitro* and *in vivo* in rats and humans. Since this peak highly correlated with the number of neural progenitor cells *in vitro*, it was suggested that this is a marker for neuronal growth. Hypothesizing that such growth is induced by ECT, Jørgensen et al. reasoned that this peak should be visible after ECT. However, Jørgensen et al. (24) were not able to validate this peak in any brain region they investigated.

## Discussion

Magnetic resonance spectroscopy gives us a unique possibility to study the biochemistry in depression and ECT. The MR scan is a gentle and non-invasive examination for the patient, demanding only that one can lay still in a noisy and cramped environment. Many investigations have been published on various substances, trying to establish a link between chemical compounds and clinical features of patients receiving ECT. Our systematic review suggests a reduction in N-acetyl aspartate following ECT, and increased choline and glutamate was also commonly reported. However, drawing firm conclusions about the effect of ECT for individual metabolites based on the MRS literature published to date is not possible based on diagnostic heterogeneity, concurrent medication use, small sample sizes, and scanner/acquisition differences. Many of the included studies have investigated both patients suffering from bipolar disorder and a unipolar disorder. Not all have published results segregated on type of depression. Yet, we might speculate that since ECT has a general effect on the brain independent of type of depression the changes we are looking for will appear in both groups. Previous reviews (18, 37, 39), as well as this one, have found that subjects with depressive episodes in general display lower values of glutamate, glutamine and/or glx, suggesting that the neurochemical changes in different types of depression are similar. Moreover, patients at different institutions follow different treatment regimens; some use medication that may affect MRS findings, voxels-placements and volumes vary, and not all of this information might be given in the published investigations. Several techniques have been used to study the metabolites of the brain in the different investigations. The majority of investigations is performed using PRESS, only one investigation not using H^1^-MRS was found. Hence, most of the investigations measured the same array of metabolites, making comparisons feasible. However, MR- spectroscopy was performed at different time points, both for patient groups and control groups. This could potentially be an advantage when comparing the fluctuation of metabolites during the course of treatment, but also makes quantitative comparisons challenging. It has also been shown that reference signal estimation for water when measuring GABA is vendor specific (40), hence complicating comparisons between different investigations.

For these reasons, and since longitudinal studies on changes after ECT investigated by MRS are not abundant, a meta-analysis based on the investigations included in this review was not considered appropriate. Bearing the above-mentioned heterogeneity in mind, there are both concordant and conflicting results of several studies, summarized below.

### NAA

Six of the eleven studies measuring NAA reported a *reduction* after ECT (20–22, 25, 26), of which one discusses that the change is due to a relative change in the reference metabolite (34). This reduction seems to normalize with time, and was not found in a long term follow up (31). Two studies reported increased levels of NAA in the ACC and the hippocampus (26, 27). Since NAA is considered a neuronal marker important for the synthesis of myelin and in general is regarded as a marker of neuronal integrity, it could be speculated that the initial reduction in NAA would be associated with the cognitive side effects. A following increase/normalization of NAA levels could explain the alleviation of some of the side effects and represent restorative processes occurring after ECT. However, this hypothesis, and whether the reduction in NAA could be associated with side effects experienced by ECT patients will need further investigation.

### Choline

All investigations examining choline reported either *increased* levels (25, 31, 32) or *no significant changes*. Choline is a marker of membrane turnover; an increase in choline values together with a reduction in NAA is found in a number of brain pathologies (11). Here, increase in Choline might reflect membrane breakdown or increased membrane turnover (11), occurring after ECT. The corresponding reduction in NAA levels is often interpreted as reduced neuronal integrity and strengthens the interpretation of increased choline after ECT as a sign of temporary disruption caused by the ECT stimulus and/or seizure.

### Myo-Inositol

Of the two investigations reporting myo-Inositol one found a significant increase (23), but only in the ACC, and not in the hippocampus. For the hippocampi, no study found a significant change in mI. As mI is considered a marker of glial function, it has been suggested that these results mark an increase of glial functioning. To conclude on the role of mI in ECT further research is warranted.

### Glutamate and Glx

Most prior studies have found that depressed subjects display lower values of glutamate/Glx (37) compared to healthy control subjects. The above-mentioned investigations comparing patients and healthy controls before ECT treatment are in accordance with these findings, but sometimes only for a subgroup of the patients, such as only unipolar depressed patients (27). We found that after treatment, glutamate or glx were either unchanged or increased (22, 25). A significant increase correlated with the treatment response of patients in two studies (27, 30). These findings suggest the involvement of glutamate in the antidepressant effect of ECT. However, in one of the studies (Njau et al.) the patients were also treated with ketamine (22), which affects the NMDA-type glutamate receptor and might be a confounder in that study.

### GABA

Only three studies investigating GABA and ECT were identified. The first investigation showed an increase in GABA levels with ECT (29), but the two other studies failed to reproduce this (20, 33). Knudsen et al. (20) tried to replicate the first investigation, by measuring GABA also in the occipital cortex, with an increased number of patients and adding a control group. Erchinger et al. (33) included 41 patients and 35 controls from two independent sites, and while no significant change in GABA after ECT was found, exploratory analyses suggested a possible link between GABA levels and side effects (effortful cognitive processing) after ECT. In summary, evidence for GABA change after ECT is sparse, and even the initial study which reported a significant increase in GABA failed to find a correlation of outcome and GABA levels (29). However, with conflicting results, more research may still be warranted, since low GABA values have previously been reported in depression (20).

### Creatine

Creatine is a marker of energy metabolism and considered relatively stable. However, some of the investigations (22, 32) showed a significant change in creatine in the course of ECT treatment. Njau (22) hypothesized an activation of the corticolimbic network, with their finding of increased creatine in the ACC. Creatine is often used as a reference metabolite, as it is more stable between scanners when compared to water (40). However, if ECT itself impacts creatine concentrations, the observed change may be driven by changes in the reference signal, rather than the metabolite of interest.

One other metabolite, showing a peak at 1.28 ppm was included in our review, due to one investigation (24) trying to replicate a marker suggested of showing neurogenesis (38). This assumption has been met with skepticism by the MRS community (41, 42), and the investigations supporting this hypothesis are few, and based on assumptions drawn from cell-cultures and animal studies. The included investigation could not find such a peak in relation to ECT, and the existence of an MRS peak reflecting neurogenesis has not been confirmed by any other investigation.

In summary, this literature review suggests changes of metabolite levels, such as reduction of NAA and increase of choline and glutamate/Glx following ECT treatment. Most studies found no change in GABA. These findings must, however, be compared with care, as the ECT procedure (including indication, administration and anesthesia, as well as time points for the MRS measurements) may vary. The current investigations may also suggest that there could be fluctuations in metabolites in the course of treatment with ECT, as several studies find *reduced* NAA in the short term after ECT followed by an increase (normalization) at later follow up time points. This is, however, difficult to explore, due to lack of detailed methodology in several of the published investigations. For most of the metabolites, such as GABA, more research is warranted before any firm conclusions can be drawn.

### Future Perspectives

MRS could have a great potential for clinical use, also in psychiatry, if metabolites predicting ECT-treatment response, outcome or cognitive side effects are to be identified. For most metabolites currently investigated no such connection could be identified, but research is heterogeneous and often published alongside other findings, as most MRS findings appear to be negative. This limits the level of detail given in published investigations, which makes it even harder to summarize the current state of MRS used on ECT patients. For many metabolites there seem to be contradicting findings, but the now accessible material cannot clearly distinguish between metabolite changes during treatment course and actual contradictions. There are also other metabolites which could be of interest, such as glutathione, which have not yet been investigated in this setting, but where both increase and decrease have been reported in depression (43). Hence, more research on already investigated and new metabolites is needed to explore the relevance and benefit of clinical use of MRS in treatment of depression. Future research focused on ECT's mechanisms of action should also investigate brain changes associated with cognitive side effects, as these side effects could parallel with neurochemical changes.

## Author Contributions

VE, LO, and LE contributed to conception and design of the investigation. VE performed the systematic search and wrote the first draft of the manuscript. All authors contributed to manuscript revision, read and approved the submitted version.

## Conflict of Interest

The authors declare that the research was conducted in the absence of any commercial or financial relationships that could be construed as a potential conflict of interest.
